# A new mechanism of action of sulodexide in diabetic nephropathy: inhibits heparanase-1 and prevents FGF-2-induced renal epithelial-mesenchymal transition

**DOI:** 10.1186/1479-5876-10-213

**Published:** 2012-10-24

**Authors:** Valentina Masola, Maurizio Onisto, Gianluigi Zaza, Antonio Lupo, Giovanni Gambaro

**Affiliations:** 1 Department of Biomedical Sciences, University of Padova, Padova, Italy; 2 Department of Medicine, Section of Nephrology, University of Verona, Verona, Italy; 3 Division of Nephrology and Dialysis, Institute of Internal Medicine and Medical Specialties, Columbus-Gemelli University Hospital, Renal Program, Catholic University, Via Moscati, 31., Rome, 00168, Italy

**Keywords:** Diabetic nephropathy, Epithelial-mesenchymal transition, Fibrosis, Heparanase-1, Sulodexide, Tubular cells

## Abstract

**Background:**

Epithelial-mesenchymal transition of tubular cells is a widely recognized mechanism that sustains interstitial fibrosis in diabetic nephropathy (DN). The signaling of FGF-2, a growth factor involved in this mechanism, is regulated by glycosaminoglycans. Heparanase-1, an endoglycosidase that cleaves heparan sulfate, is implicated in the pathogenesis of diabetic nephropathy and is necessary to FGF-2 for the induction of tubular cells transition. Well known Heparanase-1 inhibitors are heparin(s) and sulodexide, a low-molecular weight heparin – dermatan sulphate blend, which is effective in the treatment of DN.

**Methods:**

We have investigated the inhibition by sulodexide and its components of Heparanase-1 by an ELISA assay. We have analyzed its effect on the epithelial-mesenchymal transition of tubular cells by real time gene expression analysis, zymography and migration assay.

**Results:**

Results show that sulodexide is an effective heparanase-1 inhibitor, exclusively in virtue to the heparin component, with an IC50 of 5 μg/ml. In FGF-2 treated tubular cells, sulodexide also prevents the over-expression of the mesenchymal markers αSMA, vimentin and fibronectin and the motility increase, i.e. the epithelial-mesenchymal transition of tubular cells. Moreover, sulodexide prevents FGF-2 induced heparanase-1 and MMP9 increase switching off the autocrine loop that FGF-2 activates to support its signal.

**Conclusions:**

The findings highlight the capacity of sulodexide to inhibit heparanase-1 and to control tubular fibrosis triggered by epithelial-mesenchymal transition. In conclusion, these sulodexide activities support the value of this agent in controlling the progression of nephropathy to renal failure.

## Background

Diabetic nephropathy (DN) and several other chronic kidney diseases are characterized by tubular and interstitial fibrosis, which are primarily responsible for accelerating the progression to end-stage renal disease (ESRD)[1-3]. The epithelial-mesenchymal transition (EMT) of tubular epithelial cells is a process that sustains these events [4,5], and it is triggered by many factors [6-9]. A recent work of ours highlighted the central role of FGF-2 in EMT. Heparanase-1 (HPSE) is needed for EMT and by regulating syndecan-1 (SDC1) and MMP9 it sustains the FGF-2 autocrine loop [10]. HPSE is an endo-β-D-glucuronidase that cleaves heparan sulfate (HS). It takes part in extracellular matrix (ECM) remodeling and degradation, regulating the release of many HS-bonded molecules, such as growth factors, chemokines, cytokines, and enzymes, that are involved in inflammation, wound healing and tumor invasion [11,12]. A body of literature supports the involvement of HPSE in the pathogenesis of proteinuric disorders, including DN [13-15] and that is why there is great interest in identifying effective HPSE inhibitors capable of controlling mechanisms of renal damage such as EMT. The best-characterized HPSE inhibitors are low-molecular-weight heparin (LMWH) and its derivatives [11]. Previous studies have shown that sulodexide (a highly purified glycosaminoglycan [GAG] isolated from porcine intestinal mucosa, used since 1974 as an antithrombotic drug) can control proteinuria and podocyte damage by inhibiting heparanase [16-18]. Sulodexide consists for 80% of LMWH and for 20% of dermatan sulfate (DS). The heparin fraction has a molecular weight of 7000 D and a low degree of sulfation. DS is a polydisperse polysaccharide with an anticoagulant and antithrombotic activity. The treatment of DN demands additional therapeutic strategies because strict glycemic control may prove difficult to achieve in diabetic patients and, even if patients respond to conventional therapy with ACE inhibitors, kidney fibrosis slowly continues to progress and eventually leads to renal failure. It has been demonstrated that sulodexide and heparin-derived drugs are effective in the treatment of DN [19,20] and it has recently been suggested that in a rat model of peritoneal dialysis sulodexide prevents EMT in the peritoneal membrane [21]. The aim of this work was to investigate whether sulodexide inhibits HPSE, and whether this mechanism can prevent FGF-2-induced EMT in renal tubular cells. If so, sulodexide would be an interesting agent for controlling renal fibrosis and the progression of nephropathy to ESRD.

## Methods

### Heparanase assay

Twenty-five μl of matrigel (Matrigel™ Basement Membrane Matrix) at a concentration of 200 μg/ml were placed in the wells of a 96-well plate for ELISA and left to dry under an extractor hood at room temperature for 90 minutes. Test samples were prepared by mixing different concentrations of the GAGs being tested with heparanase (stabilized and lyophilized HepaOne TM Recombinant Human Haparanase-1 [rhHPA1]- InSight Biopharmaceuticals). The following GAGs were tested: sulodexide (Alfa Wassermann), the LMWH parnaparin (Alfa Wassermann), a commercial dermatan sulfate (DS) from Sigma (Sigma Aldrich C-3788), and the LMWH H2046 and dermatan sulfate D2047 (Opocrin). H2046 and D2047 are the two ingredients in sulodexide, from which they were obtained by affinity chromatography. The wells containing the matrigel were washed once with PBT (PBS+ 0.05% Tween 20) before adding the samples of enzyme/inhibitor, 25 μl per well, in working buffer (50mM Tris-HCl pH 5; 150 mMNaCl; 0.01% Triton X; protease inhibitor [complete, Roche Diagnostics]) and incubating overnight at 37°C. The heparanase enzyme was used at a concentration of 0.5 ng/μl. Each GAG was tested at four concentrations (5, 10, 20, 50μg/ml). Different GAG mixtures were tested, consisting of parnaparin with DS, and H2046 with D2047, in proportions of 20:80, 50:50 and 80:20; all GAG mixtures were tested at the same concentrations. As positive control wells were incubated overnight in working buffer.

After aspirating the treatment medium and washing with PBT 200 μl per well, the wells were saturated with blocking buffer (PBT; 0.5% BSA; 1mM EDTA) and left for 2 hours under agitation at room temperature. The blocking buffer was aspirated and the wells were washed twice with PBT. Then the samples were incubated with the primary anti-HS antibody (mouse IgM) Clone HepSS-1 (Seikagaku), 25 μl per well, diluted 1:500 in blocking buffer, for 1 hour under agitation at room temperature. Three washing cycles lasting 5 minutes each with 200 μl of PBT per well were followed by incubation for 1 hour with the secondary antibody, goat anti-mouse IgM-HRP (sc-2973, Santa Cruz Biotechnology) 25 μl per well, diluted 1:1000 in blocking buffer, under agitation at room temperature. After a further 3 washes lasting 5 minutes each with 200 μl per well of PBT, 50 μl of the ABTS (2.2’-azino-bis(3-ethylbenzthiazoline-6-sulphonic acid) liquid substrate system for ELISA (Sigma) was added to each well and the plate was kept in the dark for 15 minutes, then the reaction was blocked with 50 μl per well of 1% SDS (sodium dodecyl sulfate). The absorbance was read at 405 nm. The percentage of residual HPSE activity was calculated as follows: (max degradation – OD405 sample)/ max degradation *100.

Where max degradation = OD positive control - OD 0.5 ng/μl of heparanase in working buffer. (the addiction of GAGs at working buffer do not modify the maximal HS signal).

### Cell cultures

The human renal proximal tubular cell line, HK2 (human kidney 2), was grown in DMEM-F12 (EuroClone) (17.5 mM glucose) supplemented with 10% fetal bovine serum (Sigma Aldrich), 2 mM L-glutamine, penicillin (100 U/ml) and streptomycin (100 μg/ml), and maintained at 37°C in a 5% CO2 water-saturated atmosphere.

### mRNA expression analysis

HK2 cells were grown to subconfluence, starved in serum-free medium for 24 hours and then cultured in serum-free medium with 10 ng/ml of FGF-2 (BD Bioscience) for a further 6 hours, with or without sulodexide (50 μg/ml). Total RNA was extracted from the cells using the “GenElute Mammalian Total RNA Miniprep” kit (Sigma Aldrich). The samples were further treated with DNase (DNASE70, Sigma) to prevent any DNA contamination. The total amount of RNA and its purity were checked using the Nanodrop (EuroClone) and 1 μg of each sample was reverse transcribed into cDNA using SuperScript II Reverse Transcriptase (Invitrogen) according to the manufacturer’s instructions. Real-time PCR was performed on an ABI-Prism 7500 using Power SYBR Green Master Mix 2X (Applied Biosystems). A quantitative analysis was performed to assess the expression of fibronectin fibronectin (FN), vimentin (VIM), matrix-metalloprotease 9 (MMP9), alpha smooth muscle actin (αSMA), HPSE, SDC1. Results were normalized to glyceraldehyde-3-phosphate dehydrogenase (GAPDH) expression. The forward and reverse primer sequences have been reported elsewhere [10]. Gene expression was quantified by means of the comparative Ct method (ΔΔCt) and the relative quantification (RQ) was calculated as 2-^ΔΔCt^. Melting curve analysis was performed to check for any presence of non-specific amplification products.

### Zymography

Gelatin substrate zymography was carried out to assess the MMP-9 activity in HK2 cell conditioned media using standard procedures [22]. To obtain the conditioned media, subconfluent cells were cultured in serum-free medium for 24 h, then incubated with or without FGF-2 (10 ng/ml) and sulodexide (50 μg/ml) for a further 24 h. Equal amounts of conditioned media, obtained from the same number of cells, in sample buffer (4% SDS, 125 mM Tris-HCl pH 6.8, 20% glycerol and 0.05% bromophenol blue) were resolved in non-reducing on 10% SDS-PAGE gels copolymerized with 0.1% gelatin. After electrophoresis, the gels were washed twice for 30 min in 2.5% Triton X-100 at room temperature to remove SDS, then equilibrated for 30 min in collagenase buffer (50 mM Tris, 200 mM NaCl, 5 mM CaCl2 and 0.02% Triton X-100, pH 7.4), and finally incubated overnight with fresh collagenase buffer at 37°C. Gels were stained with 0.1% Coumassie Brilliant Blue R-250, 30% MetOH/10% acetic acid, for 1 hour and destained with 30% MetOH/10% acetic acid. Digestion bands were analyzed using the ImageJ software (http://rsb.info.nih.gov/ij/).

### Migration assay

We evaluated the migratory ability of cells in the presence of FGF-2 (10 ng/ml), and with or without sulodexide, parnaparin or DS (50 μg/ml). Briefly, a denuded area was generated on a quiescent cell monolayer of HK-2 cells by scratching with a sterile pipette tip. The monolayer was washed twice with phosphate-buffered saline (PBS) and then incubated with medium (2% FBS) containing the treatments. The cells were photographed at different time points. The scratch was measured at three points in each photo to obtain a mean value. Migration was reported as the difference (in mm^-1^) between the dimensions of the scratch at the baseline and after 24 hours [23].

## Results

### Sulodexide inhibits HPSE activity

**Figure 1:**
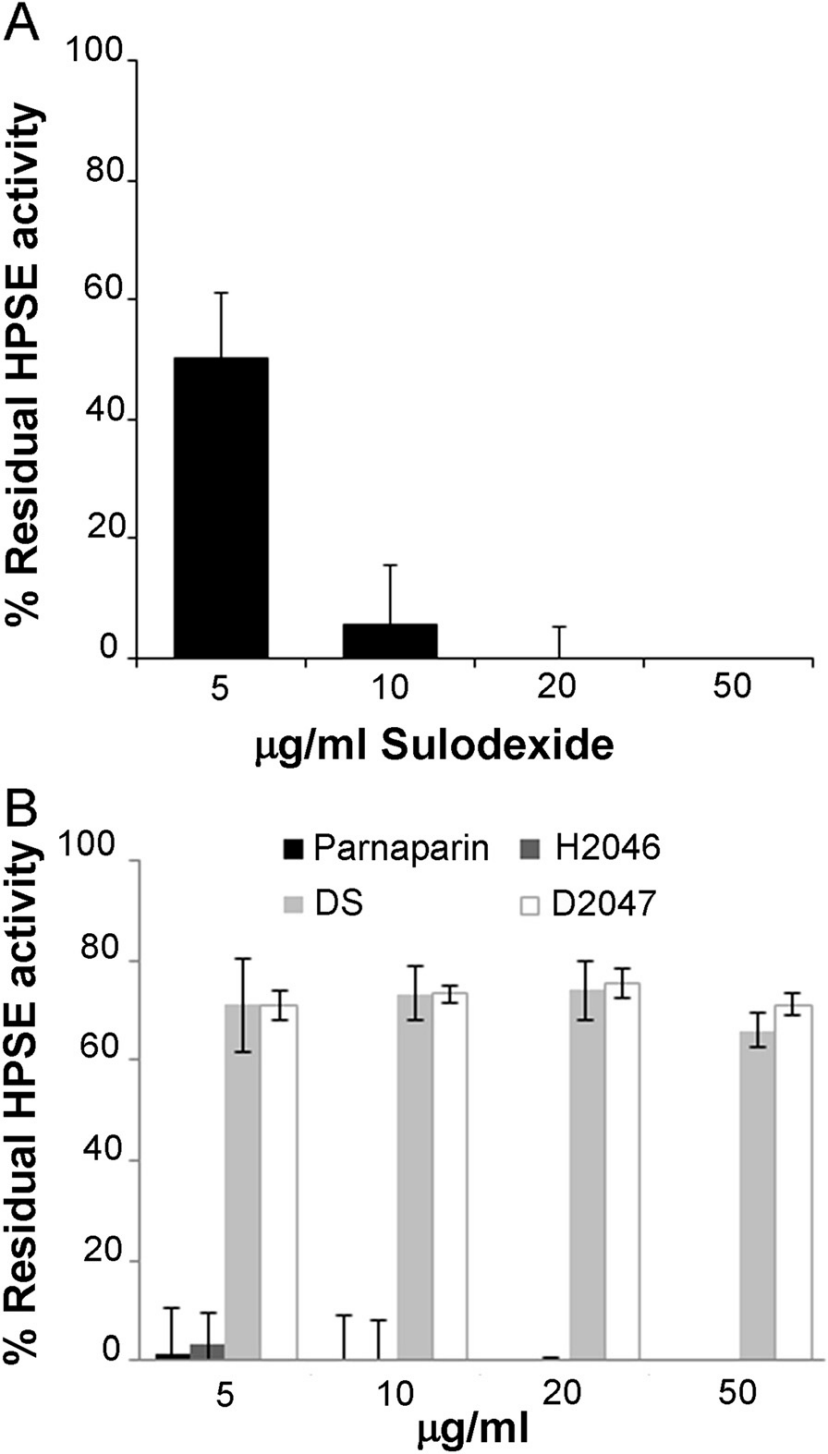
**Reduction of HPSE activity by sulodexide and GAGs.** Histograms showing the percentage of residual heparanase enzyme activity after incubation with Sulodexide and GAGs at various concentrations ± SD. All experiments were performed three times in triplicate.

Our data show that sulodexide 5 μg/ml is capable of producing a 50% heparanase inhibition; HPSE is inhibited completely with 20 μg/ml of sulodexide (Figure 1A). Since sulodexide is a mixture of LMWH and dermatan sulfate, we also analyzed the heparanase inhibiting effects of two different formulations of LMWH (parnaparin and H2046) and dermatan sulfates (DS and D2047). Parnaparin and H2046 completely inhibited heparanase at a concentration of 5 μg/ml, whereas both dermatan sulfates proved unable to reduce the enzyme activity by more than 30% at any concentration up to 50 μg/ml (Figure 1B).

**Figure 2:**
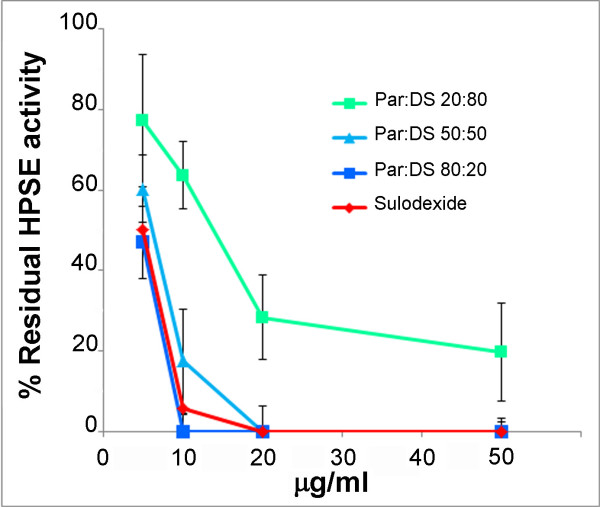
**Reduction of HPSE activity by GAG mixtures.** Graphs showing the percentage of residual heparanase enzyme activity after incubation with sulodexide and parnaparin + DS in proportions of 20:80, 50:50 and 80:20, at concentrations of 5, 10, 20 and 50 μg/ml ± SD.

To see how the relative proportions of heparin and dermatan sulfate in sulodexide could contribute to its HPSE inhibitory property, we tested H2046 + D2047, and parnaparin + DS, both in proportions of 20:80, 50:50 and 80:20. The results showed that both the 80:20 formulations completely abolished HPSE activity at a concentration of 10 μg/ml (Figure 2 compares the results for parnaparin + DS versus sulodexide).

### Sulodexide prevents any increase in mesenchymal marker expression induced by FGF-2

**Figure 3:**
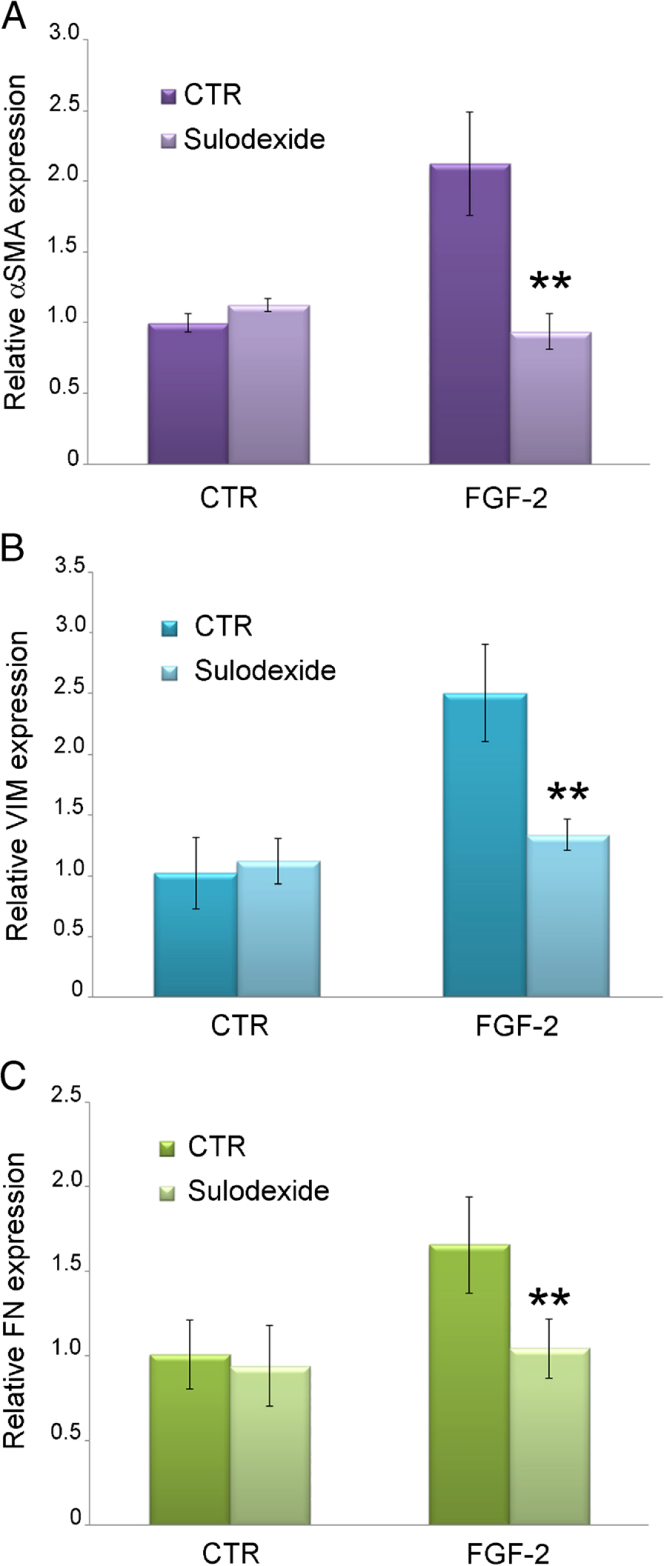
**Mesenchymal marker expression.A**) αSMA, **B**) VIM and **C**) FN gene expression in HK2 cells treated with or without FGF-2 and sulodexide. The results represent the mean ± SD of two independent experiments performed in triplicate.

HK2 renal tubular cells were starved in serum-free media for 24 hours, then treated for 6 hours with FGF-2 (10 ng/ml) with or without sulodexide (50 μg/ml). Mesenchymal marker expression was subsequently measured by real-time PCR. FGF-2 increased the expression of alpha αSMA, VIM and FN (all markers of EMT). Sulodexide did not affect the basal levels of αSMA, VIM and FN, but it completely prevented their FGF-2-induced overexpression (Figure 3).

### MMP-9 gene expression and activity

**Figure 4:**
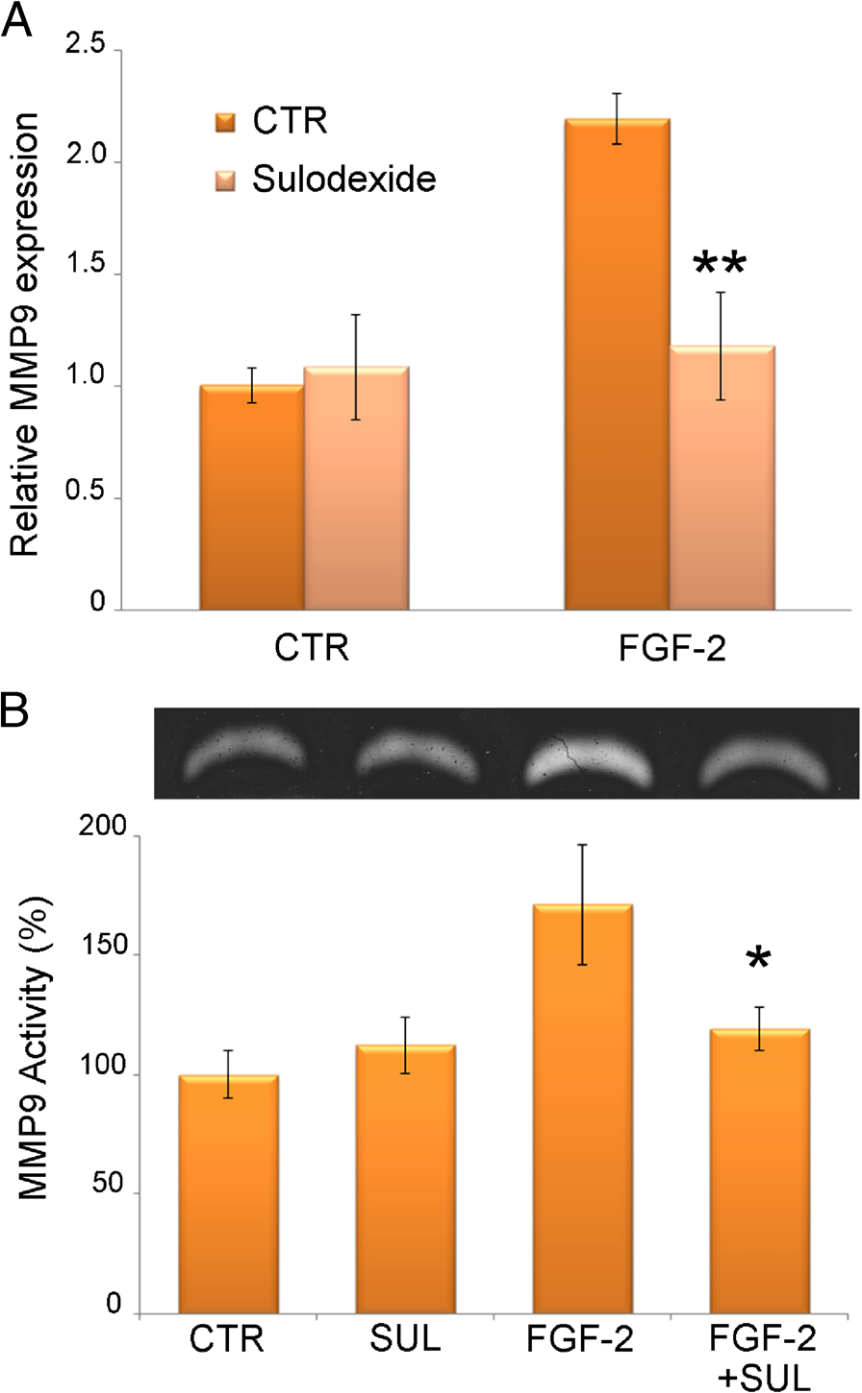
**MMP9 expression and activity.A**) MMP9 gene expression in HK2 cells treated with FGF-2 and sulodexide was measured by real-time PCR. Results represent the mean ± SD of two independent experiments performed in triplicate. **B**) *Above*, a representative gelatin zymography displaying MMP9 digestion bands produced by serum-free medium of HK2 cells cultured for 24 hours with FGF-2 and sulodexide. *Below*, densitometric analysis of MMP9 digestion bands expressed as a percentage of untreated cells. Results represent the mean ± SD of three independent experiments performed in triplicate.

Gene expression analysis showed that sulodexide prevents any increase in FGF-2-induced MMP9 gene expression without changing its basal expression level (Figure 4A). Gelatin zymography likewise confirmed that sulodexide abolished the increase in MMP9 induced by FGF-2 (Figure 4B).

### HPSE and SDC1 regulation

**Figure 5:**
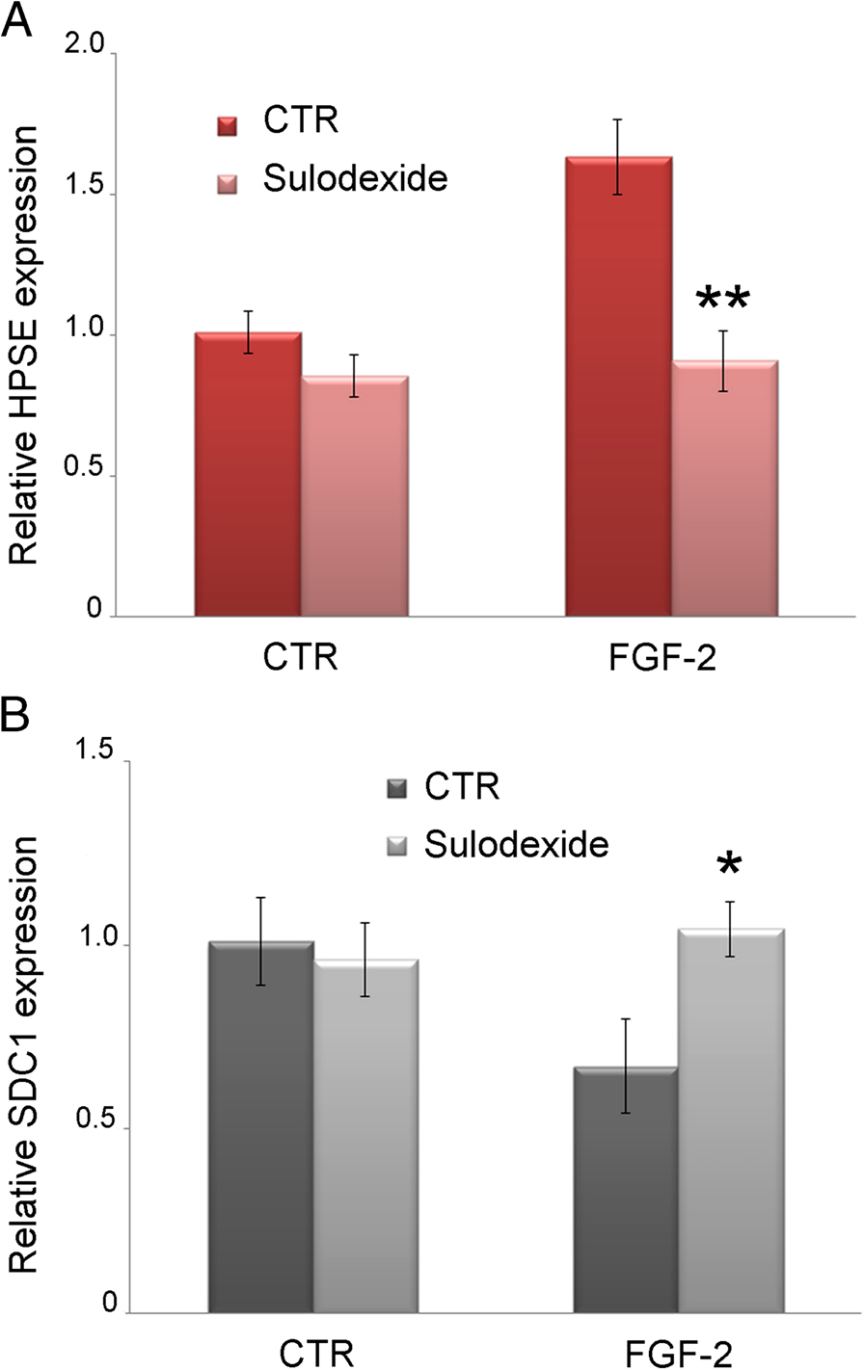
**HPSE and SDC1 gene expression.A**) HPSE, and **B**) SDC1 gene expression evaluated by real-time PCR in HK2 cells treated with or without FGF-2 and sulodexide. Results represent the mean ± SD of two independent experiments performed in triplicate.

Since we recently demonstrated that FGF-2 increases HPSE and reduces SDC1 expression, we looked into whether sulodexide could control these events. We showed that sulodexide does not affect the basal expression of HPSE and SDC1 in HK2 cells, but it does prevent the HPSE overexpression and SDC1 down-regulation induced by FGF-2 (Figure 5A and 5B ).

### Cell motility

**Figure 6:**
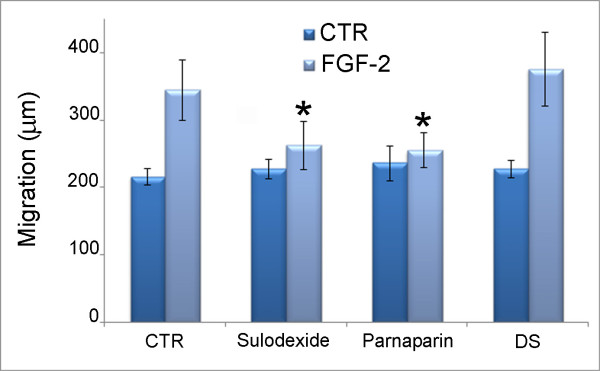
**Cell migration.** Histograms represent cell migration (in μm) over 24 hours. Data are the means of three separate experiments ± SD.

During EMT, renal tubular epithelial cells acquire a greater motility, making them better able to migrate through the basal membrane to the interstitium. FGF-2 is one of the factors triggering this event. We showed that sulodexide significantly reduced the migratory capacity of FGF-2 stimulated cells without influencing basal cell migration. We also found that parnaparin and H2046 exhibited the same behavior as sulodexide, whereas DS and D2047 were unable to inhibit HK2 cell migration (Figure 6).

## Discussion

DN occurs in up to 40% of diabetic patients and is one of the leading causes of ESRD. The approach to treating DN includes the pursuit of normoglycemia and normotension, but the search for new therapeutic strategies to prevent and treat this complication of diabetes is warranted because strict metabolic control can be difficult to achieve in many cases.

The search for new strategies includes seeking molecular targets and, in this perspective, several studies have demonstrated the involvement of HPSE in the pathogenesis of DN [14], at both tubular and glomerular levels [24] HPSE could therefore be a pharmacological target for treating DN. To date, several HPSE inhibitors have been identified, some of which are now being tested in clinical trails. Most of them are modified heparins or LMWHs [25].

GAGs like sulodexide have a favorable effect in DN. A number of mechanisms have been suggested to explain the nephroprotective effect of GAGs and sulodexide [19], including a direct inhibitory effect on HPSE [17], which reportedly increases in the glomeruli of DN patients [24]. The chemical composition of sulodexide gives the product an HPSE inhibiting action [17].

Almost all the above hypothesized mechanisms have been demonstrated at glomerular level, but one of the pathological hallmarks of the progression of kidney disease is tubulo-interstitial fibrosis. The severity of this condition has proved to be much more closely related to the risk of ESRD than glomerular lesions . The accumulation of extracellular matrix in the interstitium is sustained by the transformation of tubular epithelial cells into myofibroblasts (EMT) and this event is triggered by several growth factors and different signaling pathways [5].

We recently showed that HPSE is involved in the regulation of EMT of tubular cells induced by FGF-2. HPSE is necessary for FGF-2 to activate the PI3K/AKT pathway leading to EMT, and for FGF-2 to produce an autocrine loop by down-regulating SDC1 and up-regulating MMP9 and the same HPSE [10].

Here we demonstrate that sulodexide – a combination of GAGs composed of heparin-like (80%) and dermatan fractions (20%) that is currently used to treat thrombotic disorders and DN - is an effective HPSE inhibitor capable of preventing FGF-2-induced EMT in renal tubular cells.

Sulodexide can inhibit HPSE at therapeutic concentrations [26]: its IC50 is 5 μg/ml, and 20 μg/ml of sulodexide suffice to completely inhibit HPSE activity. Investigating the different power of the two ingredients in sulodexide, we found H2046 (and parnaparin) a very effective inhibitor of HPSE, whereas D2047 (and DS) had only a weak inhibitory action. The results of tests on combinations containing different proportions of LMWHs and dermatan sulfates confirmed that sulodexide’s HPSE-inhibiting effect is due exclusively to the heparin component, with no synergistic effect between the two ingredients.

These data are consistent with the results obtained by Naggi et al [27] using a different experimental approach. Notably, the Vlodavsky group has shown that sulodexide had a mild inhibitory effect on heparanase enzymatic activity at a concentration of 1 μg/ml, achieving a 50% inhibition with 5 μg/ml, and complete inhibition with 50 μg/ml (personal communication).

As expected, sulodexide - being an HPSE inhibitor - also prevented the overexpression of the mesenchymal markers αSMA, VIM and FN, i.e. it prevented the human renal tubular cell EMT induced by FGF-2.

Sulodexide prevented the increase in HPSE and MMP9 expression and activity and the associated SDC1 reduction that are triggered by FGF-2 in tubular cells, which means that sulodexide switched off the autocrine loop that FGF-2 activates to fuel its signal.

The fact that FGF-2 induced cell migration was inhibited by sulodexide and H2046 (and parnaparin), but not by D2047 (and DS), further confirms that sulodexide prevents FGF-2-induced EMT through its HPSE inhibiting activity.

## Conclusions

In conclusion, the present findings - together with the recent demonstration that sulodexide prevented any increase in αSMA and decrease in cytokeratin in the peritoneal membrane of a rat model of peritoneal dialysis [21] - support the conviction that sulodexide could protect against renal fibrosis sustained by EMT, thereby preventing the progression of chronic kidney disease (and DN in particular) to ESRD.

## Abbreviations

αSMA: alpha smooth muscle actin; DS: dermatan sulfate; DN: diabetic nephropathy; EMT: epithelial-mesenchymal transition; ECM: extracellular matrix; FN: fibronectin; GAPDH: glyceraldehyde-3-phosphate dehydrogenase; GAG: glycosaminoglycan; HPSE: Heparanase-1; HS: heparan sulfate; LMWH: low-molecular-weight heparin; MMP9: matrix-metalloprotease 9; SDC1: syndecan-1; VIM: vimentin.

## Competing interests

G. Gambaro has received from Alfa Wasserman SpA, Bologna, Italy, grant for research.

## Authors’ contributions

VM, MO, GZ, AL and GG designed research; VM, MO and GZ conducted research; VM, MO, GZ, AL and GG analyzed data and wrote the paper; GG had primary responsibility for final content. All authors read and approved the final manuscript.
